# Safety and efficacy of low-dose rt-PA with tirofiban to treat acute non-cardiogenic stroke: a single-center randomized controlled study

**DOI:** 10.1186/s12883-022-02808-w

**Published:** 2022-07-27

**Authors:** Zhigang Liang, Junliang Zhang, Shuangfeng Huang, Shaowan Yang, Luyao Xu, Wei Xiang, Manman Zhang

**Affiliations:** 1 Department of Neurology, Yantai Yuhuangding Hospital Affiliated to Qingdao University, 264000 Yantai, China; 2 Present Address: Yantai Yuhuangding Hostipal Affiliated to Qingdao University, No. 20 Yuhuangding East Road, Zhifu District, Shandong Province Yantai, China; 3 Binzhou Medical University, 264003 Yantai, China

**Keywords:** Low-dose rt-PA, Tirofiban, Standard dose rt-PA, Non-cardiogenic ischemic stroke, Safety

## Abstract

**Background and purpose:**

The recanalization rate after intravenous thrombolysis (IVT) is not enough and there is still the possibility of re-occlusion. We aim to investigate the effectiveness and safety of infusing tirofiban after IVT.

**Methods:**

We performed a prospective controlled study of 60 patients with acute non-cardiogenic ischemic stroke who were hospitalized in Yantai Yuhuangding Hospital from January 2018 to December 2019. The patients were divided into 2 groups: those who received tirofiban for 24 h after IVT (rt-PA + T group) and those who did not receive postprocedural intravenous tirofiban (rt-PA group). The rt-PA + T group received low-dose rt-PA (0.6 mg/kg). The rt-PA group received standard dose rt-PA (0.9 mg/kg). The main outcome measure were safety, included the symptomatic intracranial hemorrhage (sICH), any ICH, severe systemic bleeding, and mortality. The secondary outcome measure is curative efficacy which were evaluated by the 7d-NIHSS score and functional outcomes at 90 days. During hospitalization, the deterioration of neurological function was recorded.

**Results:**

All patients completed the follow-up with complete data, there were 30 patients in each of groups. The general characteristics between the two group patients had no statistically significant differences. Compared with the rt-PA + T group and the rt-PA group, in terms of safety, the rates of the sICH, severe systemic bleeding, and mortality in both groups were 0, and there was no statistically significant difference in the rates of any ICH between the two groups (10.0% vs. 3.3%, *P* = 0.306). In terms of efficacy, the rate of the early neurological deterioration events (END) was no statistical significance (0 vs. 6.6%, *P* = 0.246). There was no significant difference in the NIHSS score between the two groups before the IVT, and also at 24 h, however, the 7d-NIHSS score was lower in the rt-PA + T group compared with the rt-PA group (2.33 ± 1.85 vs. 4.80 ± 4.02, *P* = 0.004). At 90 days, 83.3% of patients in the rt-PA + T group had favorable functional outcomes compared with 60.0% of patients in the rt-PA group (*P* = 0.045).

**Conclusions:**

Low-dose rt-PA combined with tirofiban in acute non-cardiogenic ischemic stroke did not increase the risk of ICH, and mortality, and it was associated with neurological improvement.

**Trial Registration:**

The trial has been registered at the ChiCTR and identified as ChiCTR1800014666 (28/01/2018).

**Supplementary Information:**

The online version contains supplementary material available at 10.1186/s12883-022-02808-w.

## Introduction

Acute ischemic stroke (AIS) is the most common acute cerebrovascular disease, most patients may have different degrees of sequelae after the standard secondary stroke prevention, and the burden of this condition on patients, family, and society is very substantial. Intravenous thrombolysis (IVT) with recombinant tissue plasminogen activator (rt-PA) is the most effective therapy for the AIS and is recommended by clinical guidelines [1, 2], however, the recanalization rate following the IVT with rt-PA is only about 46%, and there are still about 14-34% of vascular re-occlusion in patients after recanalization [3, 4]. Therefore, enhancing the recanalization rate for clinical improvement in patients with acute ischemic stroke has always been the focus of the treatment. The tirofiban may inhibit the platelet aggregation, thus reducing the thrombus load at the site of the lesion and preventing the fibrinogen receptor from binding to the GP IIb/IIIa complex [5, 6]. Ultimately, it improves the recanalization rate of blood vessels in patients with acute ischemic stroke. In addition, the tirofiban as a reversible antagonist of the platelet GP IIb/IIIa receptor can reduce the contractile state and inflammatory response of the infarct-related blood vessels to improve the recanalization of the related blood vessels, and to improve the clinical prognosis of the patients [7]. In the studies regarding the treatment of acute coronary syndrome, it was found that the myocardial reperfusion effect of the rt-PA, when combined with tirofiban, was better than that of the rt-PA alone [8, 9]. As a result, scholars at home and abroad have continuously explored the combined treatment and found that the clinical efficacy and safety of the rt-PA and tirofiban combination in the treatment of acute ischemic stroke can be certain [10–12]. At present, 0.9 mg/kg is the recommended dose (maximum dose of 90 mg) for the IVT in China. However, Japanese Stroke Guidelines recommend a low-dose thrombolytic therapy of 0.6 mg/kg (maximum dose of 60 mg) [13, 14]. It is universally known that the similarity of platelet aggregation mechanism between the coronary atherosclerotic myocardial infarction and the atherosclerotic ischemic stroke, based on the research outcome at home and abroad and considering the safety of the study, we used low-dose rt-PA in combination with tirofiban, and this study aimed to evaluate the safety and potential efficacy of early low-dose rt-PA and tirofiban combination in patients with the non-cardiogenic ischemic stroke in a prospective single-center clinical study.

## Methods

The study was a single-center, randomized, open label, experimental study started in January 2018 and terminated in December 2019. The trial has been registered at the ChiCTR and identified as ChiCTR1800014666 on 28/01/2018. All patients randomized to study treatment will be included in a final analysis of safety and clinical outcome (intent-to-treat analysis [ITT]). The population by protocol (PP) was defined as all patients who received any dose of the study drug and met all inclusion and exclusion criteria. The data analyses of this study are available from the corresponding author upon a reasonable request.

### Study population

Sixty patients with non-cardiogenic ischemic stroke who were hospitalized (within 4.5 h of onset) in the Comprehensive Stroke Center of Yantai Yuhuangding Hospital affiliated to Qingdao University from January 2018 to December 2019 were selected as the study subjects. These patients met the diagnostic criteria for the cerebral infarction according to the Chinese Guidelines for Diagnosis and Treatment of Acute Ischemic Stroke 2018 [15]. The study protocol was approved by the medical ethics committee of Yantai Yuhuangding Hospital affiliated to the Qingdao University. The study was conducted according to the principles of the Declaration of Helsinki. All patients consented before enrollment. Additional written informed consent for the tirofiban off-label use was obtained before the use of tirofiban.

The patient was allowed to participate in the study only if he/she met all of the following criteria: (1) Age was ≥ 18 years old, (2) The patients who were hospitalized within 4.5 h after the onset of clinical symptoms, (3) The clinical diagnosis was consistent with the acute ischemic stroke, (4) There was no atrial fibrillation or severe heart disease in the past, and the ECG on the admission excluded atrial fibrillation, (5) National Institutes of Health Stroke Scale (NIHSS) score was < 15, (6) The craniocerebral CT was performed to eliminate the intracranial hemorrhage, (7) The family members agreed and signed the informed consent form. All patients who had contraindications to thrombolysis [16] or any of the criteria mentioned were excluded from the study (See Supplementary Materials).

### Data collection and assessment

After screening the AIS patients who met the inclusion criteria and the exclusion criteria, all patients were randomly divided into the rt-PA + T group and rt-PA group by a method of the table of random digit according to a ratio of 1:1. Collect demographic information and routine laboratory tests of patients. The laboratory findings included the total platelet count, cholesterol, triglyceride, low-density lipoprotein (LDL), and homocysteine (HCY) levels, and the imaging reports included bilateral carotid and vertebral artery color ultrasound images, and craniocerebral MRI plus MRA, analysing the results and providing a basis for secondary prevention at a later stage. In the rt-PA + T group, 15% of low-dose rt-PA (0.6 mg/kg) was injected intravenously within 1 min, the remaining 85% of the rt-PA was continuously pumped using a micropump over 1 h. Tirofiban (Grand Pharmaceutical Co., Ltd., Wuhan, China; National Medicine Standard; H20041165; Standard: 5 mg of tirofiban was diluted with 100 ml of normal saline) was given at the first dose of 0.4 µg/kg/min within 30 min after the IVT, then it was pumped continuously for 24 h at a dose of 0.1 µg/kg/min using a micropump. In the rt-PA group, a 10% standard dose of the rt-PA (0.9 mg/kg) was injected intravenously within 1 min, and the remaining 90% rt-PA was continuously pumped using a micropump over 1 h. After 24 h from the IVT, the patients underwent a further brain CT scan to rule out any signs of ICH, and the additional scans were performed when the patient had any signs of END (the NIHSS score has been increased by ≥ 4 points). Antithrombotic therapy was initiated if there was no ICH at 24 h after the IVT. The study considered safety as the main observational indicator, recording of bleeding events that occurred during hospitalization or within 7 days. The safety outcomes considered the sICH, any ICH, and severe systemic bleeding within 7 days or at the time of discharge. The mortality due to any cause was recorded if it occurred within 90 days after the stroke. The sICH was defined based on the European Cooperative Acute Stroke Study III study definition [17]. The ICH was defined according to the definition of the Heidelberg Bleeding Classification [18]. The secondary outcomes were efficacy, in order to objectively quantify the observational indicators, the NIHSS score and mRS score were used to evaluate the degree of clinical neurological impairment in patients, which were evaluated using the 7d-NIHSS score and 90-daysfunctional outcomes. The functional outcomes refers to the mRS scoring criteria: 0–2 points for a good prognosis, 3–5 for a poor prognosis [19].

### Statistical analysis

Previous studies have shown that the incidence of sICH was 2.4-4.9% after the IVT with the standard dose rt-PA (0.9 mg/kg) [20], the END cases have been reported to occur in 10-40% of.

patients after intravenous thrombolysis alone [21–23]. With a power of 80%, it would not be possible to detect a symptomatic hemorrhage rate below 9.7% at a 5% significance level [24], we decided to calculate the sample size by END. Considering that the occurrence of re-occlusion after intravenous thrombolysis is mostly caused by platelet aggregation [25, 26], and that tirofiban can reduce the thrombotic load at the site of lesion and reduce the occurrence of re-occlusion [27], we assuming that the incidence of END in the rt-PA + T group was 6.0%. PASS software was used for statistics, with a type I error of 0.05 on both sides, 1-β = 80%, the sample size ratio of the two groups was 1:1, group sample sizes of 23 in group rt-PA + T and 23 in group rt-PA achieve 80.3% power to detect a difference between the group proportions of 34.0%. Considering that the dropout rate was 20.0%, we calculated that the sample sizes of the two groups were 30, respectively.

The data were analyzed using the SPSS v.22.0. The data for the continuous variables were described as median, range, or mean and SD, and the categorical variables were presented as absolute and relative frequencies. The student t-test or the Mann-Whitney U test was used to compare the continuous variables, and the Pearson χ2 test or Fisher exact test was used to compare the categorical variables, as appropriate, depending on whether or not the variables were normally distributed. The Kolmogorov-Smirnov test was used to assess the normality of data distribution. The difference was statistically significant with the *P* < 0.05.

## Results

**Fig. 1:**
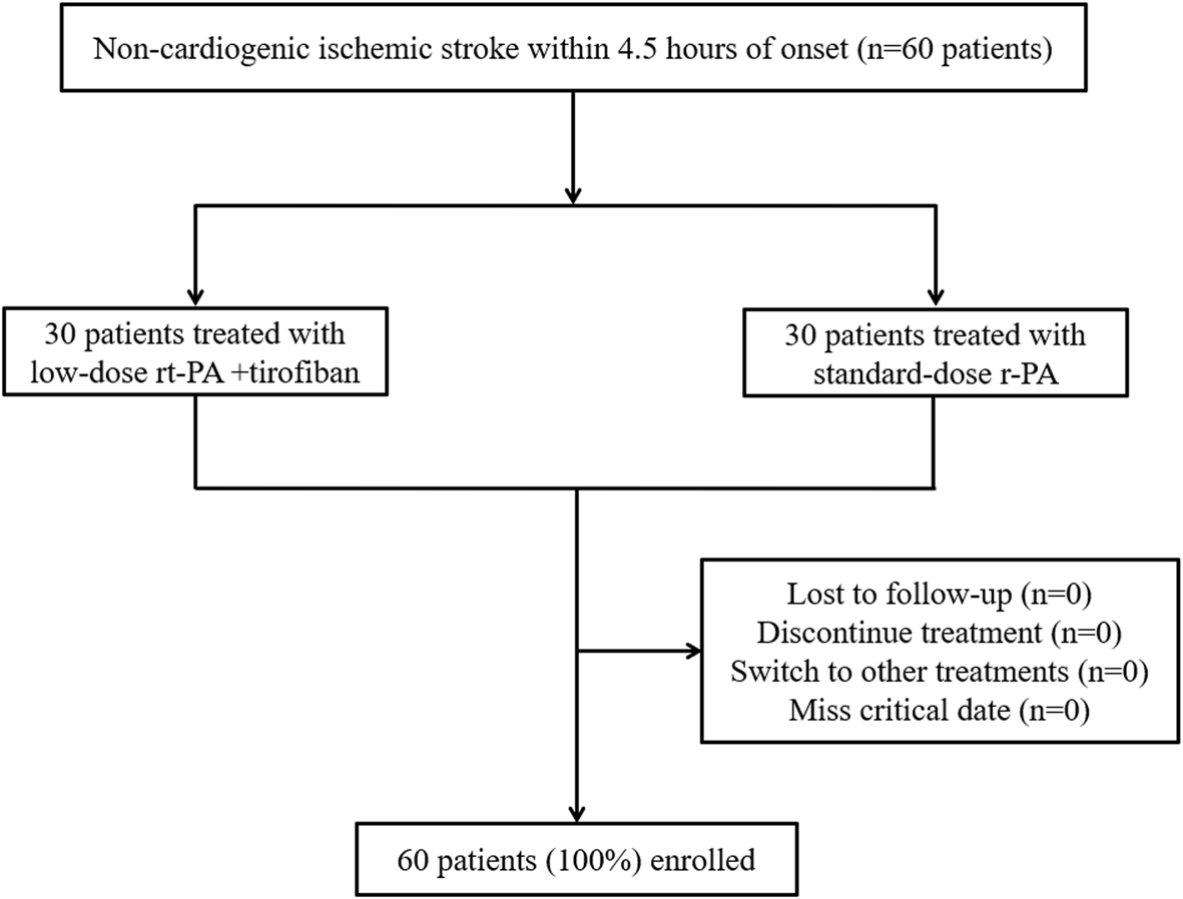
Flow Chart. All patients completed the follow-up with complete data, there were 30 patients in the rt-PA + T group and 30 in the rt-PA group

A total of 60 patients with acute non-cardiac ischemic stroke were selected with 30 patients each in the rt-PA + T group and the rt-PA group. No patients were lost to follow-up, discontinued the treatment, switched to other treatments, or missed the critical date during the study. Data for PP population were consistent with those for ITT population, and all patients completed the study (Fig. 1).

There were 20 males and 10 females in the rt-PA + T group, with an average age of 65.33 ± 8.18 years, and 18 males and 12 females in the rt-PA group, with an average age of 66.53 ± 10.27 years. There were no significant differences in smoking status, drinking habit, hypertension, diabetes, total platelets, cholesterol, triglyceride, LDL, HCY, and the NIHSS score on the time of admission between the two groups (*P* > 0.05) (Table 1).

**Table 1:** Baseline demographic and clinical characteristics

Parameter	rt-PA + T group (*n* = 30)	rt-PA group (*n* = 30)	*P*-value
Age	65.33 ± 8.18	66.53 ± 10.27	0.619
Gender(male) (n, %)	20 (66.7%)	18 (60.0%)	0.592
Smoking (n, %)	10 (33.3%)	11 (36.7%)	0.787
Drinking (n, %)	10 (33.3%)	9 (30.0%)	0.781
Hypertension (n, %)	20 (66.7%)	19 (63.3%)	0.787
Diabetes Mellitus (n, %)	7 (23.3%)	5 (16.7%)	0.519
Total platelets (x10^9^/L)	253.83 ± 11.94	249.30 ± 11.58	0.141
Total cholesterol (mmol/L)	4.71 ± 0.99	4.75 ± 1.23	0.903
Triglyceride (mmol/L)	1.29 ± 0.57	1.06 ± 0.51	0.102
LDL (mmol/L)	2.88 ± 0.79	2.88 ± 1.02	0.997
HCY (µmol/L)	11.78 ± 3.13	12.11 ± 2.70	0.670
Admission NIHSS	7.63 ± 3.54	7.37 ± 3.88	0.782

Comparing between the rt-PA + T group and the rt-PA group, the rate of the sICH, systemic bleeding, and mortality in both groups were 0, the difference was not statistically significant. Three patients in the rt-PA + T group had any ICH during hospitalization, and one patient in the rt-PA group, and there was no statistically significant difference in the rates of any ICH between the two groups (10.0% vs. 3.3%, *P* = 0.306) (Table 2).

**Table 2:** Comparison of the safety profiles between two groups

	rt-PA + T group (*n* = 30)	rt-PA group (*n* = 30)	*P*-value
sICH (n, %)	0	0	-
any ICH (n, %)	3 (10)	1 (3.3)	0.306
systemic bleeding (n, %)	0	0	-
mortality (n, %)	0	0	-

No patients in the rt-PA + T group had the early neurological deterioration events (END), and two patients in the rt-PA group, compared with the two groups, the rate of END was no statistical significance (0 vs. 6.6%, *P* = 0.246) (Table 3).

**Table 3:** Comparison of the end between two groups

Group	rt-PA + T group (*n* = 30)	rt-PA group (*n* = 30)
END (n, %)	0	2 (6.6)
No END (n, %)	30	28(93.4)
*P*-value	0.246	

**Fig. 2:**
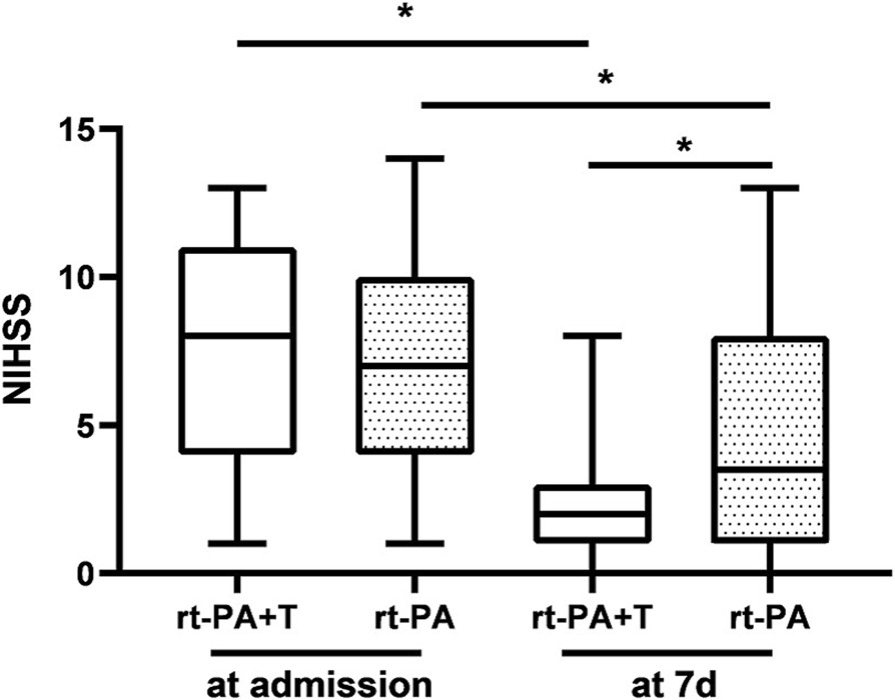
Efficacy of the outcomes at the time of admission and at 7d. The solid horizontal line indicates the median values; the box indicates the 25th to 75th percentiles, and the vertical bar indicates the 5th to 95th percentiles* *P* < 0.01, NIHSS: National Institutes of Health Stroke Scale

Compared with each time point after the treatment, it was found that the NIHSS score of the two groups showed a downward trend. There was no significant difference in the NIHSS score between the rt-PA + T group and the rt-PA group before the IVT (7.63 ± 3.54 vs. 7.37 ± 3.88; *P* = 0.782), and also at 24 h (5.57 ± 3.05 vs. 6.57 ± 4.42; *P* = 0.312). The 7d-NIHSS score was lower in the rt-PA + T group compared with the rt-PA group (2.33 ± 1.85 vs. 4.80 ± 4.02; *P* = 0.004) (Table 4). After 7 days of treatment, NIHSS scores decreased in both groups compared to pre-treatment, the rt-PA + T group is more obvious, both intra- and inter-group comparisons were statistically significant, *P* < 0.01 (Fig. 2).

**Table 4:** Comparison of the NIHSS score between the two groups

Group	No.	NIHSS score		
		Admission	24 h	7d
rt-PA + T group	30	7.63 ± 3.54	5.57 ± 3.05	2.33 ± 1.85
rt-PA group	30	7.37 ± 3.88	6.57 ± 4.42	4.80 ± 4.02
*P*-value		0.782	0.312	0.004

**Fig. 3:**
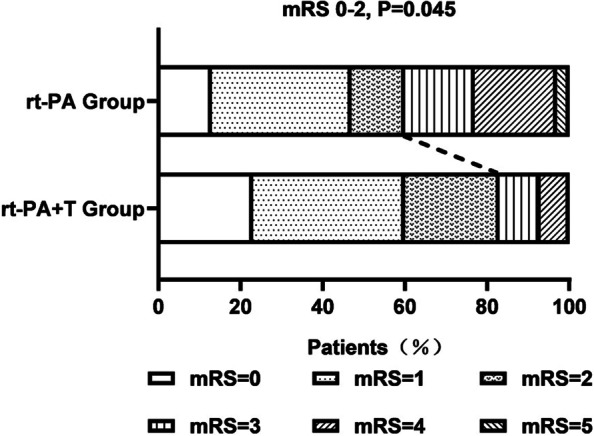
Distribution of the mRS at 90 days after the stroke. More patients in the rt-PA + T group (25/30, 83.3%) had favorable functional outcomes (mRS of 0–2) than the rt-PA group (18/30, 60.0%), *P* = 0.045

At 90 days, 83.3% of the patients (25/30) in the rt-PA + T group had a favorable functional outcome (mRS of 0–2) as compared with 60.0% (18/30) in the rt-PA group, and the difference was statistically significant (*P* = 0.045) (Fig. 3).

## Discussion

The intravenous rt-PA in patients with the ischemic stroke can achieve revascularization within the time frame(<4.5 h)of the thrombolytic therapy, restore the bloodstream of ischemic cerebral tissue, save the ischemic penumbra, and minimize the area of cerebral infarction [28]. However, the clinical effect of the rt-PA is limited. This limitation may be related to the brain ischemia caused by the delayed reperfusion [29, 30]. The rt-PA can activate the coagulation cascade in the treatment of ischemic stroke, resulting in the formation of thrombin. As the most effective platelet activator, thrombin can cause platelet aggregation and form thrombus [25]. The IVT with the rt-PA can also lead to fibrin deposition, and the activated glycoprotein (GP) IIb/IIIa receptor can further promote platelet aggregation and accumulation [26]. Based on the above mechanisms, the recanalization rate following the rt-PA treatment after the intravenous thrombolysis is only about 46%, and a considerable proportion of the patients who achieved revascularization also had re-occlusion (about 14-34%) [3, 4]. Therefore, the platelet aggregation and thrombolytic resistance of the rt-PA are the most likely causes of the recanalization failure.

Studies have shown that the use of antiplatelet aggregation drugs in the early stage of thrombolytic therapy can increase the risk of bleeding. Previous studies have also shown that the standard dose of the rt-PA (0.9 mg/kg) increases the risk of a cerebral hemorrhage in patients who were received antiplatelet therapy [31–33], the guidelines do not recommend the addition of antiplatelet drugs within 24 h of intravenous thrombolysis [34]. A recently analysis demonstrated that low-dose rt-PA was associated with significant reduction of sICH and non-inferior performance in efficacy for moderate stroke patients in China [35]. As mentioned above, there is still a high incidence of re-occlusion after intravenous thrombolysis, and it is mostly caused by platelet aggregation, so this study prospectively added tirofiban to prevent platelet aggregation after intravenous thrombolysis. In this study, in order to minimize the occurrence of the hemorrhagic transformation and ensure the safety of the research, we abandoned the standard dose of the rt-PA (0.9 mg/kg) combined with tirofiban, on the contrary, the low-dose rt-PA (0.6 mg/kg) combined with tirofiban was selected for the patients with non-cardiogenic stroke. In the study, the NIHSS scores of the two groups showed a downward trend in 24 h and 7 days after the treatment. Although the improvement in NIHSS score at 24 h was not statistically significant, it showed that in the acute phase of the stroke, the neurological function of the two groups of patients improved, and the rt-PA + T group had a more obvious improvement. At 90 days, 83.3% of patients in the rt-PA + T group had favorable functional outcomes compared with 60.0% of patients with the favorable functional outcomes in the rt-PA group (*P* = 0.045) which showed that the early tirofiban use was associated with the neurological improvement at 3 months. It is important to note that we did not test for relevant coagulation indicators (e.g. fibrinogen, total platelets) after the treatment, studies have shown that tirofiban has a short half-life and rarely causes thrombocytopenia due to the drug, with an incidence of only 0.5-2% [36], which also increases safety and reduces error in results. In addition, considering that all patients were not tested for correlation, it also reduced the bias of the results in both groups to some extent. Reviewing several previous studies [10–12, 37], we found that the results confirmed the clinical efficacy of the rt-PA and tirofiban combination in the treatment of the AIS, and this outcome was consistent with the results of this study. But to our knowledge, this study is the first to be studied on low-dose rt-pa combined with tirofiban for the treatment of non-cardiogenic ischemic stroke. Combining the results of this study, it is speculated that the tirofiban combined with the low-dose rt-PA thrombolytic therapy can improve the recanalization rate and reduce the neurological deficit at an early stage as well as improve the long-term functional outcomes.

The hemorrhagic transformation (HT) after the IVT is a pathological process of increased permeability of the blood-brain barrier caused by many factors, such as ischemic injury, reperfusion injury, and coagulation disturbance. The rt-PA and plasminogen can destroy the blood-brain barrier and interact with the matrix metalloproteinases (MMP) through the signal transduction pathways such as lipoprotein receptor-1, which aggravates the imbalance of the MMP function and accelerates the matrix degradation [38]. Studys show that fibrinogen and fibrin(ogen) degradation products (FDP) assessment are predictive factors of cerebral bleeding in rt-PA thrombolysis [39], and fibrinogen depletion increase the risk of ICH after IVT [40]. Experts say the decrease in fibrinogen plasma level after rt-PA therapy was paralleled by a significant, albeit modest, prolongation of aPTT and increase in INR, with all these changes showing strong correlations (*P*<0.001) [41]. All in all, the above interventions lead to the transformation of hemorrhage after the intravenous thrombolysis with the rt-PA. Clinical statistics show that 3–6% of the patients following the rt-PA may have HT during the thrombolytic therapy [42, 43]. The incidence of the symptomatic hemorrhage was 1.7-8.8% and the sICH was 2.4-4.9% after the IVT with the standard dose rt-PA (0.9 mg/kg), most of which occurred within the first 36 h, the sICH led to the deterioration of the neurological function and affected the outcome, as a result, the severe disability or the fatality rate of the patients was as high as 90% [20]. However, the incidence of the HT due to severe thrombocytopenia caused by tirofibanwas only 0.5–2%, it may be because the half-life of the tirofiban is about 2 h, and the prolonged bleeding time induced by the tirofiban can return to normal within 3 h after the drug withdrawal [44]. Considering the extent of fibrinogen depletion strongly depended on the dose of rt-PA [41], we used a low dose of rt-PA, which reduced the risk of bleeding. In this study, early low-dose rt-PA combined with the tirofiban used in patients did not increase the risk of the sICH, ICH, severe systemic bleeding, and mortality compared with the patients who were treated with the standard dose rt-PA. This may be related to the inclusion of more patients with mild ischemic stroke in the study and may also contribute to the high rate of good prognosis in the results. It is worthwhile to ponder that bleeding events occurred in both groups of patients, if including fibrinogen-depletion as a surrogate marker for safety of thrombolytic agents might turn useful to identify the extent of early coagulopathy and predict the risk of ICH. There was no significant difference in the incidence of the adverse events between the two groups, and this indicated that the safety of the low-dose rt-PA combined with the tirofiban in the treatment of the AIS can be guaranteed compared with the standard dose rt-PA alone, a number of previous studies had also confirmed similar result [10, 45, 46], in addition, compared to previous studies, we used low-doses of rt-PA for a higher safety profile.

However, there are several limitations of this study. First, due to the small sample size, the results need to be interpreted with caution. Second, we did not use neuroimaging to further confirm the type of arterial occlusion. Instead, we used clinical assessment as a surrogate measure, which may have room for errors. Third, due to the limitation of the time window, cardiogenic stroke was initially excluded only by electrocardiogram and previous medical history, future randomized clinical trials are warranted to validate the present results.

## Conclusions

In summary, the present study suggested that it seems to be safe and feasible to treat selected acute non-cardiogenic ischemic stroke with low-dose rt-PA followed by intravenous tirofiban. Such treatmentmay provide a new perspective for future research on acute non-cardiac ischemic stroke.

## Supplementary Information

**Additional file 1.** Supplemental Materials.

## Data Availability

The data in this study was obtained from the corresponding author upon a reasonable request, where zgliang@hotmail.com may apply.

## Abbreviations

IVT: Intravenous thrombolysis
sICH: Symptomatic intracranial hemorrhage
END: Early neurological deterioration
AIS: Acute ischemic stroke
NIHSS: National Institutes of Health Stroke Scale
mRS: Modified Rankin Scale
GP: Glycoprotein
HT: Hemorrhagic transformation
