# Supplementary material for: Safety and efficacy of low-dose rt-PA with tirofiban to treat acute non-cardiogenic stroke: a single-center randomized controlled study
Source: BMC Neurol. 2022 Jul 27;22:280. doi: 10.1186/s12883-022-02808-w (PMC9327332; doi:10.1186/s12883-022-02808-w)
Supplement: Supplementary file 1 — Additional file 1. Supplemental Materials. [file 12883_2022_2808_MOESM1_ESM.docx]

**Supplemental Materials**

Exclusion criteria: (1) Definitive diagnosis of the intracranial hemorrhage (including cerebral parenchyma hemorrhage, intraventricular hemorrhage, subarachnoid hemorrhage, subdural/ extradural hemorrhage, etc.), (2) Previous history of the intracranial hemorrhage, (3) A history of severe head trauma or stroke in the past 3 months, (4) Presence of intracranial tumors and giant intracranial aneurysms, (5) Intracranial or intraspinal surgery in the past 3 months, (6) Large surgical operations in the past 2 weeks, (7) Bleeding of the gastrointestinal or urinary system in the past three weeks, (8) Presence of active visceral hemorrhage, (9) The existence of aortic arch dissection, (10) Arterial puncture was performed in areas where it was not easy to compress hemostasis in the past week, (11) High blood pressure: systolic blood pressure of ≥ 180 mmHg or diastolic blood pressure of ≥100 mmHg after the admission, or before the thrombolytic therapy, and the blood pressure cannot be reduced to a tolerable range, (12) Blood sugar was abnormal, including blood sugar of <2.8 mmol/L or >22 mmol/L, (13) Coagulant function abnormality, there were cases where the platelet count was less than 100 × 10^9^/L or other acute hemorrhagic tendency, (14) They had been treated with the low molecular weight heparin in the past 24 hours,（15）Large infarcted area indicated by the craniocerebral CT or MRI before the thrombolysis, (16) Tirofiban was contraindicated, such as severe renal insufficiency, (17) Patients with large-vessel occlusions and underwent thrombectomy.
